# Perinatal Bisphenol A Exposure in C57B6/129svj Male Mice: Potential Altered Cytokine/Chemokine Production in Adulthood

**DOI:** 10.3390/ijerph7072845

**Published:** 2010-07-12

**Authors:** Steven D. Holladay, Shuo Xiao, Honglu Diao, Jamie Barber, Tomas Nagy, Xiaoqin Ye, Robert M. Gogal

**Affiliations:** 1 Department of Anatomy and Radiology, College of Veterinary Medicine, University of Georgia, Athens, Georgia, GA 30602, USA; 2 Physiology and Pharmacology, College of Veterinary Medicine, University of Georgia, Athens, Georgia, GA 30602, USA; 3 Interdisciplinary Toxicology Program, University of Georgia, Athens, GA 30602, USA; 4 Infectious Diseases, College of Veterinary Medicine, University of Georgia, Athens, Georgia, GA 30602, USA; 5 Pathology, College of Veterinary Medicine, University of Georgia, Athens, Georgia, GA 30602, USA

**Keywords:** bisphenol, developmental, cytokines, immune

## Abstract

Pregnant mice (n = 3) were exposed to BPA by intraperitoneal injection, from gestation day 9.5 until end of lactation. Male offspring were evaluated for cytokine production at 20 wk-of-age. One pregnant control mouse produced no males, precluding statistical analysis. However, recurring shifts in cytokines were suggested in the adult BPA offspring. Serum showed a numeric increase in 16 of 21 basal cytokine levels. ConA-stimulated splenocytes showed a numeric increase in 17 of 21 cytokines, and LPS-stimulated splenocytes an increase in 18 of 21 cytokines. The cytokine profile was one of T_H_1 up-regulation more than T_H_2, and with skewing toward T_H_17 responses.

## 1. Introduction

Bisphenol A (BPA) is a monomer component of polycarbonate plastics and epoxy resins used in numerous products of intimate human contact. These include toys, beverage containers, food packaging, medical devices, dental sealants and composites, and plastic pipes carrying household drinking water [1]. Matsumoto *et al.* [2] found BPA at detectable levels in the urine of over 90% of humans sampled, in several cases at levels comparable to those in laboratory rodents that displayed a modulation of endocrine function. Growing concerns exist over potential adverse effects that may result from BPA exposure during development, due to the transplacental transfer of the chemical [3].

Development of the immune system is highly sensitive to endocrine disrupting compound (EDC) exposure, including estrogenic chemicals. For instance, Karpuzoglu-Sahin *et al.* [4] observed permanent cytokine skewing in mice that were exposed to the non-steroidal estrogen diethylstilbestrol (DES) during gestation. BPA has activity at estrogen receptor-alpha (ERα) that is distinct from DES or estradiol, and, depending on the target tissue, may mimic, enhance, or inhibit actions of endogenous estrogen [5]. Emerging studies show BPA also binds estrogen receptor-beta (ERβ), and binds with high affinity to the non-classical membrane-bound ER (ncmER; also called estrogen-related receptor-gamma; ERRγ) and a seven-trans-membrane ER designated GPR30 [1]. When binding to classic nuclear ER, the potency of BPA is less than that of estradiol. However when the action of BPA is mediated through ncmER or GPR30, its potency can be as high as that of estradiol [6]. The present studies used a cytokine array in serum and the culture supernatants of mitogen-activated splenic lymphocytes in adult mice that had undergone perinatal exposures to BPA, to look for signs of postnatal cytokine skewing as a result of developmental exposure to BPA.

## 2. Materials and Methods

Bisphenol A (Sigma, St. Louis MO) was dissolved in sesame oil (Sigma). Wild-type C57B6/129svj mixed background mice were generated from our own colony at the University of Georgia, which itself originally derived from a colony at The Scripps Research Institute [7]. The mice were housed in polypropylene caging that does not leach BPA [8] and maintained under conditions of a 12 hr light/dark cycle, at 23 ± 1 °C with a 30–50% relative humidity. Double-distilled water was provided from a reverse-osmosis system using metal sip tubes. All methods used in this study were approved prior to study initiation by the Institutional Animal Care and Use Committee of the University of Georgia, and conformed to National Institutes of Health guidelines.

Female mice (2-3-mo-old) were mated overnight with males of similar age. The mating night was designated as embryonic day 0 (E0) if a vaginal plug was found the next morning, (which, in turn, was defined as E0.5). The plug-positive females were randomly distributed into vehicle or BPA exposure groups. Starting from E9.5, mice were intraperitoneally (*ip*) injected daily with 0 or 1.0 mg/kg/day BPA in 100 μL sesame oil. Mice were weighed daily and the amount of BPA administered was adjusted accordingly. This daily treatment lasted throughout the pregnancy and the lactation period until pups were weaned at 3 wk-of-age.

All the mice (dams and pups) were observed daily, and no overt signs of toxicity were detected. Five male offspring each from 0 mg/kg/day and 1.0 mg/kg/day-treated groups were used for analyses. These F_1_ male mice were progeny of dams as follows: 2 and 3 male offspring originated from two control dams, and 1, 2, and 2 male offspring originated from three BPA dams. Only males were studied because these mice were available from a larger male reproductive toxicity study, in which female F_1_ mice were not maintained.

The F_1_ mice were anesthetized at 20 wk-of-age by isoflurane inhalation. Blood was collected by retro-orbital bleeding after which the mice were euthanized by cervical dislocation. The blood was allowed to clot for 1 hr then centrifuged at 2,000 × g, 10 min and 23 °C, after which the serum was collected and frozen at −80 °C. Testes and epididymi were immediately collected post-euthanasia as part of the parent reproductive study. The spleen was also collected from each mouse, using dissection scissors and curved forceps. A portion of each spleen was fixed in 10% neutral buffered formalin (NBF) for histopathology, and the remaining organ was placed into pre-labeled Petri dishes (Corning, Corning, NY) containing 8 mL of RPMI-1640 culture medium (Mediatech, Herndon,VA).

The portion of each spleen not used for histopathology was gently dissociated over a stainless steel sieve screen (Sigma) using curved forceps. Cells were then pipetted through the screen following dissociation to remove debris. The cells were then washed in RPMI-1640 by centrifugation for 10 min (at 240 × g and 23 °C). The supernatant was then discarded and the cell pellet was re-suspended in 1 mL incomplete RPMI-1640. To each tube, 2 mL of ACK (*i.e.*, 0.83% [v/v] ammonium chloride) lysis buffer (pH 7.29) was added to lyse red blood cells, and the tubes were incubated for 5 min at 23 °C. After the lysis incubation, the cells were re-suspended in 5 mL of incomplete RPMI-1640 and washed twice by centrifugation for 7 min (at 290 × g and 7 °C). The leukocyte-rich cells were then re-suspended in 5 mL complete RPMI-1640 media containing 10% fetal bovine serum (FBS; Sigma), 2 mM L-glutamine (ICN, Costa Mesa, CA), 50 IU penicillin/mL [ICN], and 50 mg streptomycin/mL [ICN]), and maintained on ice until culturing.

Splenic cells at 5.0 × 10^6^/mL were cultured for 48 hr in 6-well plates with either 10 μg/mL of the T-cell mitogen Concanavalin A (Con A) or 25 μg/mL of the B-cell mitogen lipopolysaccharide (LPS), at 37 °C and under 5% CO_2_. Following this incubation, the plates were centrifuged, and the supernatants collected and frozen at −80 °C until time of the cytokine assay.

The expression of 21 cytokines/chemokines was evaluated using a Multiplex™ MAP Mouse Cytokine/Chemokine assay (Millipore, Boston, MA), according to the manufacturer’s protocol. Briefly, 25 μL of serum and 25 μL of supernatant from the 48 hr-cultured ConA- and LPS-stimulated splenocytes were aliquoted (along with appropriate standards) into duplicate wells of a pre-wet Millipore filter plate containing 25 μL of assay buffer. To each well, 25 μL of premix cytokine/chemokine beads were added, after which the plate was sealed and placed on an orbital shaker for 18 hr at 4 °C. The plate was washed with buffer and incubated with 25 μL/well of detection antibody for 60 min at 23 °C. This step was followed by the addition of 25 μL/well of Strepavidin-PE, and a further incubation for 30 min at 23 °C. The plate was washed with wash buffer, after which the wells were re-suspended with 150 μL/well of sheath fluid and counted on a Luminex 200 (Luminex Corporation, Austin, TX) and analyzed with XPONENT software solutions (Luminex). The results were reported in pg/mL.

## 3. Results

The profile of cytokine response in the five control mice (from n = 2 dams) and the five BPA-exposed mice (from n = 3 dams) was one of generalized enhancement of cytokine production after developmental exposure to BPA. In serum, 16/21 cytokine levels were numerically greater in the perinatally-exposed BPA mice (Table 1). For their ConA-stimulated spleen cells, this number was 17/21 (Table 2), and for LPS-stimulated spleen cells this was 18/21 (Table 3). Several cytokine levels were doubled, or greater, in the different compartments in mice that received the BPA exposures.

## 4. Discussion

Bisphenol A (BPA), widely used for production of plastics, was recently found in maternal and fetal serum, follicular fluid, and amniotic fluid at 1.0–8.3 ng/mL [9]. The sensitivity of the developing immune system to agents that bind the estrogen receptor (ER) raised questions about possible contributions of early BPA exposure to human immune-mediated diseases later in life. The importance of the androgen receptor [10] and aryl hydrocarbon receptor in immune development [11] further suggests the potential for BPA to induce developmental immunotoxicity.

The present male mice were made available to our laboratory as unutilized extra mice from a larger study. Because the dam is the statistical unit, insufficient mice were available to detect more than potential trend immune effects from the perinatal exposure to BPA. Our laboratory was equipped to run the cytokine analysis and so elected to do so, in order to determine if suggestive data may result.

The cytokine/chemokine levels in the perinatally BPA-exposed mice, compared to levels in the controls, showed a general pattern of up-regulation in all three bio-sample sets evaluated. The serum from the BPA-exposed mice showed an approximate doubling of basal levels of pro-inflammatory innate cytokines, interleukin (IL)-1β, G-CSF, and GM-CSF. Levels of G-CSF and GM-CSF were again approximately doubled in the supernatants of splenocytes from these exposed hosts when stimulated with the T-lymphocyte mitogen Con A. These data may suggest a functional skewing of T-lymphocytes and some degree of active inflammation. For example, G-CSF plays a major role in neutrophil production and survival and at elevated levels has been reported to exacerbate existing inflamatory disease in humans and mice [12].

Levels of IL-17 were also approximately doubled in supernatants of BPA-treated splenocytes stimulated with Con A, and approximately tripled as a result of LPS stimulation. The primary role of T_H_17 cells and their cytokines, e.g., IL-17, IL-21, and IL-22, is to mediate host defense to various infections. Inappropriate over-activity of these cells is involved in the pathogenesis of many autoimmune diseases [13,14].

The present data were collected in five mice per exposure, however the statistical unit (dam) was smaller, at two controls and three BPA-treated. Nonetheless, cytokine/chemokine skewing due to perinatal BPA exposure is clearly suggested in the array data. Further studies with larger group size are needed to determine if the present trend data represent a real effect, in that changes of the sort suggested may relate to increased risk of immune-mediated diseases in the offspring of exposed pregnant women.

## Figures and Tables

**Table 1:** Cytokine/chemokine levels in serum after perinatal BPA exposure.

**Cytokine or Chemokine**	**Control Serum (pg/mL)**	**BPA Serum (pg/mL)**
**G-CSF**	**541.4 ± 58.2**	**1311.4 ± 315.4**
**GM-CSF**	**3.4 ± 0.3**	**6.8 ± 2.0**
**IFNγ**	24.6 ± 4.3	21.0 ± 2.7
**IL-10**	8.8 ± 1.6	11.3 ± 0.9
**IL-12p70**	**9.8 ± 1.7**	**16.0 ± 2.1**
**IL-13**	13.0 ± 1.9	12.6 ± 1.4
**IL-15**	17.0 ± 1.5	20.4 ± 1.0
**IL-17**	15.6 ± 3.1	18.5 ± 5.0
**IL-1α**	**56.7 ± 5.7**	**104.6 ± 18.4**
**IL-1β**	**4.5 ± 0.7**	**8.4 ± 0.4**
**IL-2**	5.0 ± 1.0	5.0 ± 0.8
**IL-4**	4.1 ± 0.5	5.1 ± 0.5
**IL-5**	42.5 ± 2.5	40.0 ± 7.7
**IL-6**	72.6 ± 29.6	66.9 ± 14.3
**IL-7**	8.5 ± 1.3	9.9 ± 0.8
**IP-10**	170.9 ± 25.8	217.9 ± 47.4
**KC**	76.9 ± 31.0	98.8 ± 27.4
**MCP-1**	12.5 ± 1.6	14.7 ± 2.0
**MIP-1α**	5.0 ± 0.6	5.1 ± 0.5
**Rantes**	**117.9 ± 25.5**	**203.2 ± 43.1**
**TNFα**	**5.1 ± 0.8**	**7.9 ± 2.0**

Levels numerically increased by ≥33% are shown in bold. Numbers are presented as means ±SEM.

**Table 2:** Cytokine/chemokine levels in supernatants from ConA-stimulated splenocytes after perinatal BPA exposure.

**Cytokine or Chemokine**	**Control (pg/mL)**	**BPA (pg/mL)**
**G-CSF**	**711 ± 70**	**1,384 ± 73**
**GM-CSF**	**4,460 ± 1174**	**9,017 ± 1360**
**IFNγ**	20,930 ± 872	20,767 ± 1297
**IL-10**	192 ± 42	212 ± 61
**IL-12p70**	**90 ± 4**	**150 ± 19**
**IL-13**	73 ± 7	93 ± 10
**IL-15**	61 ± 5	50 ± 11
**IL-17**	**5,943 ± 550**	**11,018 ± 1,528**
**IL-1α**	162 ± 5	209 ± 18
**IL-1β**	16 ± 1	21 ± 4
**IL-2**	12,246 ± 1269	13,374 ± 1781
**IL-4**	**253 ± 34**	**424 ± 102**
**IL-5**	4,939 ± 953	3,366 ± 992
**IL-6**	**3,941 ± 434**	**5,266 ± 1129**
**IL-7**	37 ± 3	40 ± 3
**IP-10**	9,031 ± 242	8,777 ± 610
**KC**	454 ± 46	510 ± 98
**MCP-1**	1,332 ± 262	1,654 ± 396
**MIP-1α**	3,984 ± 886	3,684 ± 894
**Rantes**	9,852 ± 377	10,011 ± 137
**TNFα**	**974 ± 182**	**1,307 ± 190**

Levels numerically increased by ≥33% are shown in bold. Numbers are presented as means ±SEM.

**Table 3:** Cytokine/chemokine levels in supernatants from LPS-stimulated splenocytes after perinatal BPA exposure.

**Cytokine or Chemokine**	**Control (pg/mL)**	**BPA (pg/mL)**
**G-CSF**	7,198 ± 778	8,819 ± 1,658
**GM-CSF**	**73 ± 13**	**245 ± 62**
**IFNγ**	**966 ± 376**	**2,318 ± 1,097**
**IL-10**	605 ± 94	515 ± 167
**IL-12p70**	39 ± 4	45 ± 5
**IL-13**	41 ± 5	46 ± 5
**IL-15**	67 ± 5	69 ± 5
**IL-17**	**83 ± 9**	**315 ± 199**
**IL-1α**	356 ± 47	445 ± 74
**IL-1β**	29 ± 7	28 ± 4
**IL-2**	93 ± 12	97 ± 6
**IL-4**	10 ± 1	12 ± 1
**IL-5**	34 ± 5	31 ± 7
**IL-6**	5,677 ± 561	6,256 ± 233
**IL-7**	55 ± 12	59 ± 5
**IP-10**	8,512 ± 895	8,801 ± 627
**KC**	892 ± 87	1111 ± 87
**MCP-1**	735 ± 171	822 ± 261
**MIP-1α**	5,736 ± 632	7,434 ± 1,205
**Rantes**	9,852 ± 377	10,011 ± 137
**TNFα**	980 ± 97	1,155 ± 140

Levels numerically increased by ≥33% are shown in bold. Numbers are presented as means ±SEM.
